# First Evaluation of Drug-Resistant *Mycobacterium tuberculosis* Clinical Isolates from Congo Revealed Misdetection of Fluoroquinolone Resistance by Line Probe Assay Due to a Double Substitution T80A-A90G in GyrA

**DOI:** 10.1371/journal.pone.0095083

**Published:** 2014-04-17

**Authors:** Alexandra Aubry, Wladimir Sougakoff, Pamela Bodzongo, Guy Delcroix, Sylvie Armand, Gérald Millot, Vincent Jarlier, René Courcol, Nadine Lemaître

**Affiliations:** 1 UPMC Université, Paris, France; 2 INSERM U1135, Paris, France; 3 Centre National de Référence des Mycobactéries et de la Résistance des Mycobactéries aux Antituberculeux, Paris, France; 4 Faculté des Sciences de la Santé, Brazzaville, Congo; 5 Laboratoire de Bactériologie-Hygiène du CHRU, Lille, France; 6 Université Lille Nord de France, Lille, France; 7 UDSL, F-59000 Lille, France; 8 INSERM U1019-UMR8204, Lille, France; St. Petersburg Pasteur Institute, Russian Federation

## Abstract

**Background:**

Tuberculosis (TB) is one of the major public health problems in Congo. However, data concerning *Mycobacterium tuberculosis* drug resistance are lacking because of the insufficient processing capacity. So, the aim of this study was to investigate for the first time the resistance patterns and the strain lineages of a sample of *M. tuberculosis* complex (MTBC) isolates collected in the two main cities of Congo.

**Methods:**

Over a 9-day period, 114 smear-positive sputa isolated from 114 patients attending centers for the diagnosis and treatment of TB in Brazzaville and Pointe Noire were collected for culture and drug susceptibility testing (DST). Detection of mutations conferring drug resistance was performed by using line probe assays (GenoType MTBDR*plus* and MTBDR*sl*) and DNA sequencing. Strain lineages were determined by MIRU-VNTR genotyping.

**Results:**

Of the 114 sputa, 46 were culture positive for MTBC. Twenty-one (46%) were resistant to one or more first-line antiTB drugs. Of these, 15 (71%) were multidrug resistant (MDR). The most prevalent mutations involved in rifampin and isoniazid resistance, D516V (60%) in *rpoB* and S315T (87%) in *katG* respectively, were well detected by MTBDR*plus* assay. All the 15 MDR strains were susceptible to fluoroquinolone and injectable second-line drug. No mutation was detected in the *rrs* locus involved in resistance to amikacin and capreomycin by both the MTBDR*sl* assay and DNA sequencing. By contrast, 9 MDR strains belonging to the same cluster related to T-family were identified as being falsely resistant to fluoroquinolone by the MTBDR*sl* assay due to the presence of a double substitution T80A-A90G in GyrA.

**Conclusions:**

Taken together, these data revealed a possible spread of a particular MDR clone in Congo, misidentified as fluoroquinolone resistant by MTBDR*sl* assay. Thus, this test cannot replace gold-standard culture method and should be interpreted carefully in view of the patient's native land.

## Introduction

Tuberculosis (TB) remains an important cause of morbidity and mortality in 2012 with 8.6 million cases and 1.3 million deaths worldwide [1]. Sub Saharan Africa is particularly affected with approximately 1.3 million new cases. Congo is a West Africa country with a population of ∼4 million people. As in many sub Saharan Africa countries, TB is a major health problem in Congo despite the implementation of the Directly Observed Therapy Short course (DOTS) strategy in the mid-1990. The World Health Organization estimated the incidence of all forms of TB in Congo at 381/100,000 and mortality at 42/100,000 in 2012 [1]. Among the new TB cases, the rate of HIV patients reaches 33%. Thus, Congo is considered a “high burden” country for TB and HIV infection. The Global Plan to STOP TB (2006–2015) recommends knowing early the drug susceptibility status, especially in case of retreatment. During the past few years, several molecular techniques including reverse hybridization assay with DNA probes have been developed to quickly predict drug resistance in clinical isolates of *Mycobacterium tuberculosis* complex (MTBC) and thereby to facilitate appropriate patient management. GenoType MTBDR*plus* demonstrated a good concordance with phenotypic drug susceptibility testing and has been approved by the WHO for rapid screening of patients at risk of MDR-TB. Unfortunately, routine drug susceptibility testing (DST) is not implemented in Congo due to the lack of laboratory capacity for mycobacterial culture, and molecular methods targeting drug resistance mutations. As in other African countries, smear microscopy is the primary method used for diagnosing TB in Congo, and if diagnosed, a standardized supervised treatment is set, with the following regimens: 2HRZE/4HR and 2HRZES/1HRZE/5HRE (H: isoniazid, R: rifampin, Z: pyrazinamide, E: ethambutol and S: streptomycin) for new and previously treated TB cases, respectively. Consequently, to date, no study has been conducted in this country to reflect the situation in term of resistance phenotypes and molecular mechanisms of resistance. This study represents the first phenotypic and molecular study of tuberculosis in Congo with the goal to (i) determine drug susceptibility patterns in samples collected in the two main Congo's cities (Brazzaville and Pointe-Noire) that account for two third of the population and (ii) characterize mutations prevalent in clinical isolates by using the commercially available GenoType MTBDR*plus* and GenoType MTBDR*sl* assays, as well as DNA sequencing [2], [3]. Furthermore, to gain an insight into the epidemiology of these MTBC isolates, a molecular typing by determination of the MIRU-VNTR patterns was also performed. Based on the results obtained, we report for the first time the presence of MDR but not XDR strains in Congo. We also showed that reverse hybridization assay with DNA probes falsely detected fluoroquinolones resistance in a predominant MDR clone harboring a double substitution T80A-A90G known to confer hypersusceptibility to fluoroquinolones [4], [5], [6]. This emphasized that detection of *gyrA* mutations by line probe assay should be interpreted with caution and cannot replace conventional drug susceptibility testing in countries such as Congo.

## Materials and Methods

### Ethics statement

Ethical approval for this study was obtained from the Faculty of Sciences de la Santé of Congo review committee. Informed written consent was waived by the IRB because all data and sampling were collected during the course of routine care in patients registered at the DOTS centers for suspicion of pulmonary TB or TB treatement. This study did not require any additionnal data, sampling and contact with patients. The outcome of the research data did not affect patient management. The objectives of the study were verbally explained to all participants. They were free to refuse that their sputum was transported to TB reference laboratories in France for processing and culture. No personal identifiers were recorded to maintain anonymity and confidentiality.

### Patients and specimens

One smear-positive sputum per patient was collected over a 9-day period from 114 patients attending 6 centers for the diagnosis and treatment of TB of the two largest cities of Congo (5 located in Brazzaville and one located in Pointe Noire). Samples were studied anonymously in combination with the available clinical information including sex age, HIV status and past history of treatment. All patients included in this study were previously untreated or treated for less than a month at the time of collection of smear-positive sputum.

### Culture and antibiotic susceptibility testing

The 114 refrigerated sputa were shipped to the laboratory of bacteriology from the teaching hospital at Lille (France) for culture and determination of first-line drug susceptibility of isolates as well as mutations profiles associated with the resistance to antiTB drugs. Samples were processed by standard methods (N-acetyl L-cysteine-NaOH), followed by staining with auramine-rhodamine fluorochrome for microscopy and by culturing in a bactec MGIT 960 (Becton-Dickinson) non radiometric automatic isolation system [7]–[8]. Primary culture was characterized as belonging to MTBC using an immunochromatographic assay, based on detection of a specific MPT64 antigen (BIOCENTRIC, France). When the immunochromatographic assay was negative, identification of mycobacteria was done by 16S-23S rDNA internal transcribed spacers or Hsp65 sequencing [9], [10]. A total of 46 samples were culture positive for MTBC and were used for determining the drug susceptibility profiles against the first-line drugs: isoniazid (0.1 µg/ml), rifampin (1.0 µg/ml), ethambutol (5 µg/ml) and streptomycin (1 µg/ml) by using the MGIT 960 system according to the manufacturer's instructions [11]. Isolates resistant to at least to isoniazid and rifampin were considered as multidrug resistant (MDR) isolates and underwent drug susceptibility testing with ofloxacin, moxifloxacin and amikacin to identify extensively drug resistant (XDR) TB isolates. The breakpoint concentrations of ofloxacin, moxifloxacin and amikacin with MGIT 960 system were 2, 0.5 and 1 µg/ml, respectively [12], [13]. Two concentrations of ofloxacin and moxifloxacin were tested (1 and 2 µg/ml, and 0.25 and 0.5 µg/ml, respectively).

### DNA extraction

For DNA preparation, 1 ml of bacteria cultured in MGIT was used. Liquid cultures were centrifugated at 10,000 g for 15 min. and the mycobacterial pellets were resuspended in 300 µl distilled water and heat-killed at 95°C for 30 min. DNA used for amplification by PCR was obtained by heat shock extraction (3 cycles of freezing at −80°C and boiling for 1 min.)

### Detection of mutations in drug target genes in MTBC isolates

In order to screen for resistance to rifampin and isoniazid, the GenoType MTBDR*plus* assay was performed as recommended by the manufacturer (Hain LifeScience, GmbH, Germany). In addition, MTBC isolates found to be MDR were tested by GenoType MTBDR*sl* assay, a line probe assay (LPA) that detects mutations associated with fluoroquinolone and injectable second-line drugs resistance. DNA sequencing in specific hot-spot target regions of the following genes: *rpoB* (rifampin resistance), *katG, inhA* and *inhA* promoter region (isoniazid resistance), *gyrA, gyrB* (fluoroquinolone resistance), *rpsL* and *rrs* (aminoglycoside resistance) was performed to characterize more precisely the pattern of mutation or to resolve discrepancies between LPA and DST. The primers used for the DNA sequencing are listed in Table 1. The PCR products were purified using a Macherel-Nagel PCR purification kit according to the manufacturer's instructions. The purified DNA was used for DNA sequencing with a 3500 Dx Genetic analyzer (Applied Biosystem) automated sequencer.

**Table 1:** Designed primers used in this study for amplification of drug target genes.

Locus	Primers	Sequence (5′ to 3′)	Position	Size (pb)
promoter *inhA*	pro-F inhA	TCAATACACCCGCAGCCA	−189	491 pb
	pro-R inhA	GTCATCCGCATGAGGAAT	302	
*inhA*	inhA-F	GCATGGGTATGGGCCACT	−38	528 pb
	inhA-R	GACCGTCATCCAGTTGTA	490	
*katG*	katG1-F	GCCCGAGCAACACCCACC	3	705 pb
	katG2-R	TTCGGCCCCTCCGGGTTC	708	
	katG3-F	CAGTGGGAGCCCGATGAG	568	721 pb
	katG4-R	GCGGCCCAAGGTATCTCG	1289	
	katG5-F	CGCTCCCCGACGATGCTG	1117	783 pb
	katG6-R	GACGCGCAGGCCACCTAC	1900	
	katG7-F	CGTGCTGGAGCCCAAGGC	1755	446 pb
	katG8-R	GGTTCATCACCTTGTCCC	2201	
*rpoB*	rif-F	GCGTACGGTCGGCGAGCT	1361	419 pb
	rif-R	CCTTGCGGTACGGCGTTT	1780	
*gyrA*	gyrA-F	CGATTCCGGCTTCCGCCC	180	195 pb
	gyrA-R	CGCCGGTGGGTCATTGCC	375	
*gyrB*	gyrB-F	GAGTTGGTGCGGCGTAAG	1390	321 pb
	gyrB-R	CGGCCATCAGCACGATCT	1711	
*rpsl*	rspl-F	CCAACCATCCAGCAGCTG	4	305 pb
	rspl-R	ATCCAGCGAACCGCGGAT	309	
*rrs*	rrs3-F	TGGGTTAAGTCCCGCAAC	1077	459 pb
	rrs3-R	AAAGGAGGTGATCCAGCC	1536	

### MIRU-VNTR typing

The MTBC culture positive isolates were further genotyped by PCR amplification of a panel of 24 MIRU-VNTR loci at the National Reference Centre for Mycobacteria (Pitié-Salpêtrière hospital, Paris, France), as previously described by Supply *et al*. [14]. Briefly, 2 µl of DNA extracted from cultures of the MTBC isolates was amplified using the 24 loci MIRU-VNTR typing kit (Genoscreen, Lille, France). Automated MIRU-VNTR analysis was performed on an ABI 3730 automatic sequencer (Applied Biosystems, California, USA). Estimation of the sizes of the amplified fragments was done using the Genemapper software (Applied Biosystems) with automated assignment of the alleles. Identification of the families and lineages was carried out by using the MIRU-VNTR plus online database.

### Statistical analysis

Epidemiological and laboratory analysis were done with EpiInfo (version 6.04). Associations between variables were explored using the X^2^ test (or Fisher's exact test when expected cell sizes were less than five). A *p* value less than 0.05 was considered statistically significant.

## Results and Discussion

Of the 114 recruited cases, 81 and 33 were registered in the DOTS centers of Brazzaville and Pointe Noire, respectively. From the 114 sputa, a total of 46 and 5 cultures were positive for MTBC and *M. avium*, respectively. The rate of culture positive for MTBC was significantly higher (*p* = 0.002) in Pointe Noire (21/33, 64%) than in Brazzaville (25/81, 31%) while the rate of patients treated at the time of collection of smear-positive sputa (66% *vs* 60%, *p* = 0.53) as well as the delay between collection of specimens and the time of initiation of supervised treatment (means  =  8.4±8.8 *vs* 11.0±11.8 days, *p* = 0.25) did not differ significantly among patients originating from these two cities. Thus, the treatment with the first-line drugs of TB cases from Pointe-Noire area appeared less effective than in Brazzaville. Finally, 47 (41%) were culture negative and 16 (41%) were contaminated by oral flora.

A high frequency of resistance (21/46, 46%) to one or more first-line antiTB drugs was noted in the MTBC isolates tested for susceptibility with the MGIT 960 system (Table 2). Among the 21 resistant strains, 15 (71%) were MDR, 4 were isoniazid monoresistant, one was streptomycin monoresistant and one was resistant to both isoniazid and streptomycin. As expected, MDR was more significantly prevalent in the 9 patients with previous history of TB (78% *vs* 22%, *p* = 0.003) since all the 7 resistant strains isolated from the retreatment cases were MDR (Table 2). Alarmingly, the remaining 8 MDR-TB cases (53%) were found in the new cases suggesting a recent transmission of MDR-TB. The rate of MDRTB was comparable to the one observed in the hot-spot countries located in Eastern Europe and also in some countries of Africa such as Sierra Leone and Cameroon [1], [15], [16] and, largely exceeded the latest WHO's global estimation (3.1%). It is worth noting that the neighboring countries such as RD of Congo and Cameroon reported an increase in MDR-TB in recent years thus suggesting a possible spread of MDR-TB strains in this region of Africa [1], [16]. The MDR-TB cases were not associated with HIV infection in the present study. By contrast, the proportion of MDR was significantly higher in culture-positive patients attending the DOTS center of Pointe-Noire compared to patients from the Brazzaville district (52% *vs* 16%, *p* = 0.02) thus, indicating a possible outbreak in the Pointe Noire area.

**Table 2:** MTBC clinical isolates susceptibilities to antimicrobial drugs.

	Sensitivity to all first-line drugs	Resistant to at least one drug but not to INH and RIF	MDR
New cases of TB	23	6	8
Recurrent or relapse cases of TB	2	0	7*

*p = 0.003 (patients with previous history of TB *vs* no previous history of TB).

Rifampin resistance was only observed in MDR isolates. The hybridization patterns obtained with the MTBDR*plus* assay confirmed the resistance to rifampin detected by DST in all isolates, either by a positive hybridization signal with the MUT1 (RpoB substitution D516V) or MUT3 (RpoB substitution S531L), or the lack of hybridization with the wild-type probes WT2, WT3, WT4 and WT8. Nine of 15 MDR strains harbored the RpoB substitution D516V, whereas the RpoB substitution S531L commonly detected in MDR strains worldwide isolated, was found in two strains only [17], [18]. All these substitutions were confirmed by *rpoB* DNA sequencing, which revealed an additional V577M substitution in a strain also harbouring the S531L substitution. Four remaining MDR strains, detected by the lack of hybridization with the wild-type probes WT2, WT3 and WT4 harbored rarely encountered *rpoB* mutations as shown by DNA sequencing: D516Y, S531Q, and double mutations L511R+D516Y and L511P+D516Y. In a previous study, single substitution at position 511 (L511P) and 516 (D516Y) were associated with discordant drug susceptibility results [19] while in the present study the combination of these substitutions were detected in highly rifampin-resistant strains.

Considering the 20 isoniazid-resistant strains, the hybridization patterns obtained with the MTBDR*plus* assay confirmed the resistance to isoniazid detected by DST in all, except 3 isolates. The most prevalent S315T substitution was found in 15 (75%) isolates both with the genotype MTBDR*plus* assay and DNA sequencing [17], [18]. Mutations at the *inhA* position -15 (C→T) in the promoter site, corresponding to *inhA* MUT1 band, were only detected in two non MDR isolates which were characterized by a low level of resistance to INH. The three isoniazid-resistant strains not detected with MTBDR*plus* assay included two MDR strains exhibiting a H270P mutation and a large deletion of *katG* in the 5′ part of the gene, respectively and one monoresistant to INH having a single *katG* missense mutation at codon 709 (E → K). Resistance to streptomycin which was not detectable by LPA assays was due to the presence of substitution K43R in *rpsL* of three MDR and one non MDR strains. Then, the 15 MDR isolates were screened for the identification of a XDR profile by DST to fluoroquinolones (ofloxacin and moxifloxacin) and injectable second-line drug (amikacin), and by genotypic MTBDR*sl* assay. All the 15 MDR strains were susceptible to amikacin and fluoroquinolones thus excluding the presence of XDR strains in the Congo's isolates studied. The 15 MDR strains phenotypically susceptible to amikacin also harbored a wild-type nt 1400 region of *rrs*. In contrast, 9 of the 15 MDR strains, predicted by MGIT 960 to be susceptible to ofloxacin and moxifloxacin, were detected as potentially resistant to fluoroquinolones by MDRTB*sl* assay by lack of hybridization with the WT2 probe while no hybridization was observed with all the mutation probes (MUT1, MUT2, MUT3 A→D) thus, indicating an unusual mutation in *gyrA*. A possible resistance to fluoroquinolone suggested by the MDRTB*sl* results but remaining undetectable at the phenotypic level was unexpected since the second-line drug-based regimen has not yet been introduced in Congo. DNA sequencing of *gyrA* revealed that these 9 MDR strains harbored the same combination of GyrA T80A and A90G substitutions known to confer hypersusceptibility to fluoroquinolones [4]–[6]. We also sequenced *gyrB* because mutations in this gene that have been previously associated with fluoroquinolone resistance are not explored by the MTBDR*sl* assay [20]. One MDR strain displayed the substitution G559A in GyrB but this mutation is not involved in fluoroquinolone resistance [21]. In our study, a GyrA T80A and A90G substitutions were also found in one strain susceptible to first-line drugs and the GyrB G559A substitution in three non MDR strains: two drug susceptible and one isoniazid monoresistant strains (data not shown). GyrA T80A and A90G and GyrB G559A substitutions may represent natural variations of *M. tuberculosis* resulting in false-resistance to fluoroquinolones by LPA which can lead to a XDR-TB misdiagnosis and unnecessary restriction of active drugs. Natural variations in the genome especially in the *gyrA* gene at position 73, (T73A) and 95 (S95T) have been previously reported in MTBC isolates from other African countries [22], [23] and emphasized that LPA assay should be used and interpreted with caution in patients originating from these countries. Finally, second-line DST may be more accurate for the detection of second drugs in these resource-limited TB settings. For this purpose, recent studies highlighted the usefulness of the sensititre MycoTB plate method in low-income countries such as Tanzania and Uganda [24], [25]. This method is rapid (the turnaround time is 10–15 days when the test is valid on the first try), ready to use (lyophilized first and second-line drugs with several dilutions), and requires minimal instrumentation (mirror box) [26]. This method could be useful for DST surveillance in Congo especially among patients receiving empirical MDR regimen that could be introduced in this country because of the absence or low-prevalence of fluoroquinolone and injectable aminoglycoside resistance.

Analysis of the dendrogram drawn from the MIRU-VNTR codes confirmed that the 9 MDR strains exhibiting the double mutation GyrA T80A and A90G, as well as the same mutations in *rpoB* and *katG*, were gathered into a unique cluster related to the T-family. All except one of the 9 strains displayed an identical MIRU-VNTR. The last strain differed at the level of one locus among the 24 loci analyzed (Table 3) thus, suggesting a possible focal outbreak in Pointe Noire district since 6 strains of this clone were isolated from patients attending the DOTS center of Pointe-Noire. The one susceptible strain harboring the double mutation in *gyrA* was also related to the T-family too but was not included in the same cluster encompassing the nine T80A-A90G MDR strains. One MDR strain and the three non MDR strains displaying the GyrB G559A substitution were linked in the MIRU-VNTR dendrogram and were related to the T lineage suggesting that this substitution could be a marker of a subgroup of strains in the T family. Only 4 MTBC (3 MDR and one susceptible) isolates belonged to the Beijing family in our study. A similar low prevalence of Beijing family among MTBC isolates was previously reported in the West Africa countries [15], [27], [28], contrarily to Asia, Russia and the United States where the Beijing family predominates [29]–[32]. The 3 MDR Beijing strains that were closely related at the level of their MIRU-VNTR profile (Table 3) shared a D516Y substitution in RpoB and two of these had additional *rpoB* mutations at position 511. The two remaining MDR strains characterized by a S531 substitution in RpoB, were found to be related to the LAM family but had unique MIRU-VNTR codes, indicating that they were not related to any of the other MDR isolates (Table 3). Finally, MIRU-VNTR analysis revealed that a majority of the MTBC isolates included in our study (30 of 46, 65%) were related to the ill-defined T family which represents a group of strains showing a high degree of genetic diversity frequently encountered in Africa. The other genotypes found in the 46 strains analyzed were LAM (17%), Cameroon (9%) and Beijing (9%). The distribution of MIRU-VNTR families was similar between strains isolated in Pointe Noire and Brazzaville areas and between MDR and non MDR strains. No significant statistical link was found between lineages and epidemiological data such as age, sex and HIV status.

**Table 3:** Characteristics of mutations in drug target genes from the 15 MDR-TB isolates.

No. of isolates	Drug target genes					MIRU-VNTR patterns	Spoligotyping based defined clades
	*rpoB*	*katG*	*rpsL*	*gyrA*	*gyrB*		
N = 8	D516V	S315T	wt	T80A+A90G	wt	253333253232237253214423	T
N = 1	D516V	S315T	wt	T80A+A90G	wt	254333253232237253214423	T
N = 1	S531L+V577M	H270P	wt	wt	G559A	255333143262325262213423	T
N = 1	D516Y	S315T	K43R	wt	wt	273335444432455253213423	Beijing
N = 1	L511R+D516Y	S315T	K43R	wt	wt	273335444432655253213423	Beijing
N = 1	L511P+D516Y	S315T	K43R	wt	wt	273335444432655253213423	Beijing
N = 1	S531L	S315T	K43R	wt	wt	251313542122437262112415	LAM
N = 1	S531Q	Del	wt	wt	wt	251323142122234262213523	LAM

## Conclusions

By conducting the first phenotypic and molecular investigation of *M. tuberculosis* clinical isolates in circulation in Congo we have revealed the presence of MDR-TB in this country as well as the probable spread of a particular clone of double mutant GyrA T80A and A90G hypersusceptible to fluoroquinolones among MDR strains in this region of Africa. The presence of MDR-TB emphasizes the urgent need to perform molecular assays to rapidly detect drug resistance in order to start an appropriate antiTB treatment with second-line drugs in Congo. However, line probe assay may have limitation in detecting fluoroquinolone resistance in Congo since strains harboring specific mutations such as T80A-A90V in GyrA lead to false diagnosis of fluoroquinolone resistance. Therefore, a lack of hybridization with the wild type WT2 probe should not be interpreted as fluoroquinolone resistance until DNA sequencing and/or DST were performed.

## Acknowledgments

The authors thank the entire staff of the centers for the diagnosis and treatment of TB of Brazzaville and Pointe-Noire for their help in this study.
