# Correction: First Evaluation of Drug-Resistant *Mycobacterium tuberculosis* Clinical Isolates from Congo Revealed Misdetection of Fluoroquinolone Resistance by Line Probe Assay Due to a Double Substitution T80A-A90G in GyrA

**DOI:** 10.1371/journal.pone.0113219

**Published:** 2014-11-05

**Authors:** Nadine Lemaître

The original Ethics Statement for this article contained incorrect information regarding approval of this study. The correct information is provided in this revised Ethics Statement:

“The Ethics Committee for the protection of humans participating in Sciences of Government of the Republic of Congo (CERSSA) exempted this study from review and waived the need of informed written consent due to the fact that all data and sampling were collected during the course of routine care in patients registered at the DOTS centers for suspicion of pulmonary tuberculosis or tuberculosis treatment. Authorization for the research was given by the Minister of Health and Population and the General Director of the National Public Health Laboratory from the Republic of Congo. This study did not require any additional data, sampling and contact with patients. The outcome of the research data did not affect patient management. The objectives of the study were verbally explained to all participants. They were free to refuse that their sputum was transported to TB reference laboratories in France for processing and culture. No personal identifiers were recorded to maintain anonymity and confidentiality.”

The Dean of the Faculty of Health Sciences of Marien Nagoubi has issued a formal statement confirming that the need for ethics approval was waived for this study, and that the study was authorized by the Minister of Health and Population and the General Director of the National Public Health Laboratory of Congo. This statement can be viewed below in file S1.

File S1 **Waiver of Ethics Approval from the Dean of the Faculty of Health Sciences.**
(PDF)
