# Supplementary material for: Correction: First Evaluation of Drug-Resistant Mycobacterium tuberculosis Clinical Isolates from Congo Revealed Misdetection of Fluoroquinolone Resistance by Line Probe Assay Due to a Double Substitution T80A-A90G in GyrA
Source: PLoS One. 2014 Nov 5;9(11):e113219. doi: 10.1371/journal.pone.0113219 (PMC4221480; doi:10.1371/journal.pone.0113219)
Supplement: File S1 — Waiver of Ethics Approval from the Dean of the Faculty of Health Sciences. (PDF) [file pone.0113219.s001.pdf]

UNIVERSITE MARIEN NGOUABI
-=-=-=-=-=-=-=-=-=-=-
FACULTE DES SCIENCES DE LA SANTE
-=-=-=-=-=-=-=-=-=-=-=-
B.P. 2672- Brazzaville – CONGO
-=-=-=-=-=-=-=-=-=-
CABINET DU DOYEN
-=-=-=-=-=-=-=-
Courriel : fssa_cg@yahoo.fr

Travail * Progrès * Humanité
-=-=-=-=-=-=-=-=-=-

Brazzaville, le 6 OCT 2014

V/Réf. :
N/Réf. : № 275

Dear Editor,

To whom it my concern

I undersigned, Pr Jean-Rosaire IBARA, Dean of the Faculty of Health Sciences of Marien Ngouabi, certify that the study reported in the manuscript untitled "First Evaluation of Drug-Resistant Mycobacterium tuberculosis Clinical Isolates from Congo Revealed Misdetection of Fluoroquinolone Resistance by Line Probe Assay Due to a Double Substitution T80A-A90G in GyrA" (PLoS ONE 9(4): e95083. doi:10.1371/journal.pone.0095083) was waived from submission to the Ethics Committee for the protection of humans participating in Sciences of Government of the Republic of Congo (CERSSA) by the office of the Dean of the Faculty of Health Sciences for two reasons :
(i) the samples were routinely collected from patients seeking treatment for tuberculosis at dedicated treatment centers in Brazzaville and Pointe-Noire and no additional data collection occurred for this study.

(ii) the Minister of Health and Population and the General Director of the National Public Health Laboratory of Congo authorized this study that was the thesis project of now Doctor Pamela Bodzongo.

Consequently, a partnership between the Faculty of Health Sciences of Marien Ngouabi and the University of Lille (Bacteriology Laboratory of teaching hospital of Lille) was established for culture of samples and drug resistance pattern determination. The samples were collected between the 7 and 14th July 2010 in DOTS centers of Brazzaville and Pointe-Noire by Mrs BODZONGO Pamela who was subsequently welcomed (from July to September 2010) in the Bacteriology Laboratory of teaching hospital of Lille for routine processing of samples.
I authorize PlosOne to publish this letter with the corrections of Ethical statements paragraph.

Sincerely yours

Professor Jean-Rosaire IBARA
